# Associations between Adenotonsillar Hypertrophy, Age, and Obesity in Children with Obstructive Sleep Apnea

**DOI:** 10.1371/journal.pone.0078666

**Published:** 2013-10-25

**Authors:** Kun-Tai Kang, Chen-Han Chou, Wen-Chin Weng, Pei-Lin Lee, Wei-Chung Hsu

**Affiliations:** 1 Department of Otolaryngology, National Taiwan University Hospital, Taipei, Taiwan, R.O.C.; 2 Department of Otolaryngology, Taipei Hospital, Ministry of Health and Welfare, New Taipei City, Taiwan, R.O.C.; 3 Institute of Epidemiology and Preventive Medicine, College of Public Health, National Taiwan University, Taiwan, R.O.C.; 4 Sleep Center, National Taiwan University Hospital, Taipei, Taiwan; 5 Department of Pediatrics, National Taiwan University Hospital, Taipei, Taiwan, R.O.C.; 6 Department of Internal Medicine, National Taiwan University Hospital, Taipei, Taiwan, R.O.C.; National Taiwan University Hospital, Taiwan

## Abstract

**Objective:**

To investigate the contributions of adenoid and tonsil size to childhood obstructive sleep apnea (OSA) and the interactions between adenotonsillar hypertrophy, age, and obesity in children with OSA.

**Methods:**

In total, 495 symptomatic patients were recruited. The patients were assigned to four groups according to age：toddler (age 1-3, n=42), preschool (age 3-6, n=164), school (age 6-12, n=200), and adolescence (age 12-18, n=89). All subjects had tonsil size graded by otolaryngologists, adenoid size determined on lateral radiographs (Fujioka method), and a full-night polysomnography. The apnea-hypopnea index (AHI), adenoid size, and tonsil size were compared in obese and non-obese children in the four age groups. Adjusted odds ratios (ORs) and 95% confidence interval (CI) of adenotonsillar hypertrophy and OSA risk were estimated by multi-logistic regression.

**Results:**

The AHI was positively related to tonsil grade (r=0.33, p <0.001) and adenoid size (r=0.24, p <0.01) in all patients. Tonsil grade was positively related to AHI in all four age groups. Adenoid size was positively related to AHI in the toddler, preschool, school groups, but not in the adolescent group (r=0.11, p=0.37). Tonsil grade and adenoid size were both positively related to AHI in obese and non-obese children. In the regression model, obesity (OR=2.89; 95% CI 1.47-5.68), tonsillar hypertrophy (OR=3.15; 95% CI 2.04-4.88), and adenoidal hypertrophy (OR=1.89; 95% CI 1.19-3.00) significantly increased OSA risk.

**Conclusions:**

Adenotonsillar hypertrophy and obesity are the major determinants of OSA in children. However, the influence of adenoid size decreases in adolescence.

## Introduction

Obstructive sleep apnea (OSA) in children is a respiratory disorder characterized by upper airway collapse during sleep [1-3]. Untreated OSA is associated with adverse cardiovascular [4], neurocognitive [5], and somatic growth consequences [6]. Adenotonsillar hypertrophy is the major determinants of OSA in children. Removing the tonsils and adenoids is widely recognized as the most effective first-line therapy for childhood sleep apnea [7,8]. However, scientific studies regarding the correlations between adenotonsillar size and polysomnographic features remain diverse and controversial [9-11]. Nolan et al. [9] reviewed studies relating tonsil size to OSA and found a weak association between subjective pediatric tonsil size and objective OSA severity. Major et al. [10] and Feres et al. [11] both described methodological disparities and inadequacies in adenoid size assessments in current data.

The upper airway morphology is largely influenced by adenotonsillar and facial growth patterns that display discrepancies in OSA children of different ages and levels of adiposity [12,13]. Therefore, the magnitude of adenotonsillar effects on childhood OSA may be altered by age and obesity [14,15]. Tagaya et al. [14] asserted that the correlation between adenoid size and OSA is more prominent in preschool children than in school-aged children. Dayyat et al. [15] reported a modest association between adenotonsillar sum scores and apnea index in non-obese children, but not in obese children. However, the relationships between adenotonsillar size and OSA in detailed age groups, and the respective effect of adenoid and tonsil size on OSA in obese and non-obese children, have not been well investigated.

The main purposes of this study were to (1) critically examined the respective correlations between adenoidal size, tonsil size, and OSA in children in detailed age groups (i.e., toddler, preschool, school, and adolescent) and different levels of adiposity (i.e., obese and non-obese), and (2) further elucidate the respective contributions of adenoidal hypertrophy and tonsillar hypertrophy to childhood OSA. We hypothesize that the effects of adenoid size and tonsil size on OSA differ among children in different groups.

## Materials and Methods

### Basic Data

This retrospective study were approved by the Ethics Committee of the National Taiwan University Hospital. The informed consent form was not obtained, because all the data were analyzed anonymously and it specifically waived by the approving IRB. Children under 18 years of age with OSA-related symptoms were recruited from the respiratory, pediatric, psychiatric, and otolaryngologic clinics of the hospital between May 2010 and January 2012. Children younger than 12 months were first excluded from our study because sleep patterns and obstructive sleep disorders were different in subjects younger than 1 year of age. Exclusion criteria were (1) suboptimal sleep studies (total sleep time <4 hours, or sleep efficiency <60%) (2), cranio-facial anomalies (3), genetic disorders, neuro-muscular diseases, cognitive deficits, or mental retardation (4), children younger 12 months of age, and (5) previous tonsil, adenoid, or pharyngeal surgery. Detailed histories were obtained and physical examinations were carried out. Basic data, including age, gender, symptoms and signs of sleep disturbances, were recorded, as were history of nasal allergy, otitis media with effusion, and sinusitis or asthma.

The remaining children were carefully assessed for their demographics, clinical symptoms, and physical evaluations. Though children were recruited from different clinics, only children clinically suggestive of OSA, such as the presence of snoring or witness of apnea, were sent for a overnight polysomnographic sleep study to quantify the presence and severity of OSA. Children with complete demographic data and sleep studies were acquired for inclusion into our study.

Subjects were divided into four age groups: toddler (age 1-3), preschool (age 3-6), school (age 6-12), and adolescence (age 12-18) [16-18]. The weight and height of each child were measured and body mass index (BMI) was calculated. The age and gender corrected BMI was applied using established guidelines [19]. Obesity was defined as a BMI higher than the 95^th^ percentile for a child’s age and gender [20].

### Assessments of adenoid and tonsil size

Adenoid size was determined based on a lateral cephalometric radiographs, which was obtained by the radiology department at National Taiwan University Hospital using standard techniques. The adenoidal-nasopharyngeal (AN) ratio was measured on the lateral radiograph as the ratio of adenoidal depth to nasopharyngeal diameter according to the method by Fujioka et al. [21] These measurements were acquired by an investigator who was blind to the sleep study results. Adenoidal hypertrophy was considered when the AN ratio was higher than 0.67 [22,23].

The tonsils were graded using the scheme by Brodsky et al. [24]: Grade I) small tonsils confined to the tonsillar pillars; grade II) tonsils that extend just outside the pillars; grade III) tonsils that extend outside the pillars, but do not meet in the midline; grade IV) large tonsils that meet in the midline. Tonsillar grade were assessed by an investigator who was blind to the aim of the study. Tonsillar hypertrophy was defined as grade III or above [9,24].

### Polysomnography (PSG)

Full night PSG (Embla N7000, Medcare Flaga, Reykjavik, Iceland) was performed in a sleep lab following the established protocol [22,25-27]. The sleep stage and respiratory events were scored according to the standard of the American Academy of Sleep Medicine [26]. Obstructive apnea was defined as the presence of continued inspiratory effort associated with a >90% decrease in airflow for duration of ≥ 2 breaths. Hypopnea was defined as a ≥50% decrease in airflow for duration of ≥ 2 breaths associated with arousal, awakening, or reduced arterial oxygen saturation of ≥ 3%. All sleep studies were analyzed by the same investigator, who was blind to the aim of the study, to maximize inter- and intra-scorer reliability. Diagnosis of pediatric OSA was defined as the presence of an apnea/hypopnea index (AHI) ≥ 1 event per hour in the overnight polysomnographic study [7,8,27-29].

### Statistical analysis

Data were analyzed using SPSS version 17.0 (SPSS Inc., Chicago, IL, USA). Continuous data are expressed as mean plus standard deviation, and categorical data as number and percentage. The correlations between adenoid size and AHI, or tonsil size and AHI in all participants as well as in age groups and adiposity were analyzed using a Pearson’s correlation. To explore the correlations between adenoidal hypertrophy and tonsillar hypertrophy, all participants were divided into four additional groups: subjects without adenotonsillar hypertrophy; with adenoidal hypertrophy; with tonsillar hypertrophy; and with adenotonsillar hypertrophy. The OSA risk for these four groups was analyzed by logistic regression. Finally, the associations between demographics and OSA risk were calculated using a multivariable logistic regression model. A p value less than 0.05 was considered statistically significant.

## Results

### Study population

Initially, 815 subjects were identified for possible inclusion. Fifty-seven children were excluded due to incomplete records or suboptimal PSG data, and 103 children were excluded due to co-morbidities that met exclusion criteria. Another 160 subjects had previous tonsil, adenoid, or pharyngeal surgeries. In total, 495 subjects were enrolled into the final analysis.

Mean age of study participants was 7.9±4.2 years. Boys comprised 69.5 % (344/495). Forty-two were toddlers (1-3 years), 164 were pre-school age (3-5 years), 200 were school age (6-12 years), and 89 were adolescents (13-18 years). Eighty-two children were obese, and 413 were non-obese; One hundred three subjects had no adenoidal or tonsillar hypertrophy; 93 subjects had adenoidal hypertrophy only, 127 subjects had tonsillar hypertrophy only; and 172 subjects had adenotonsillar hypertrophy.

Mean AHI was 8.4±11.6 events/hour for children with tonsillar hypertrophy, and 3.1±6.7 events/hour for those without tonsillar hypertrophy. Mean AHI was 7.7±11.4 events/hour for children with adenoidal hypertrophy, and 3.3±6.8 events/hour for those without adenoidal hypertrophy. Mean AHI was 10.4±13.3 events/hour for obese children, and 4.8±8.6 events/hour for non-obese ones. For age groups, the mean AHI was 4.3±7.1 events/hour for toddlers, 6.0±9.6 events/hour for pre-school children, 5.6±10.1 events/hour for school-aged children, and 6.2±10.3 events/hour adolescents.

### Association between adenoid size, tonsil size, and obstructive sleep apnea

**Figure 1:**
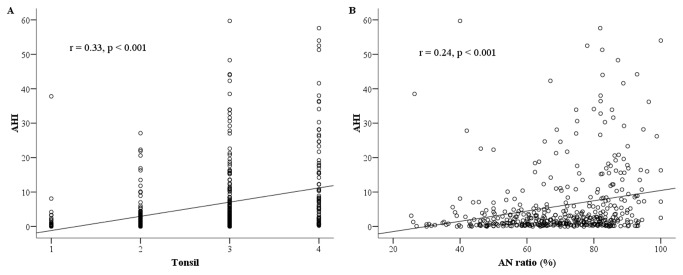
Scatterplot of tonsil grade, AN ratio and AHI for all subjects. (**a**) Scatterplot of tonsil grade and AHI for all subjects. (**b**) Scatterplot of AN ratio and AHI for all subjects. *Note*: AN ratio = adenoidal-nasopharyngeal ratio; AHI = apnea/hypopnea index.

 For all participants, a positive linear relationship existed between tonsil grade and AHI as a measure of OSA severity (r=0.33, p <0.001) (Figure 1a). Similarly, a positive relationship existed between adenoid size (AN ratio) and AHI for all subjects (r=0.24, p <0.001) (Figure 1b).

**Figure 2:**
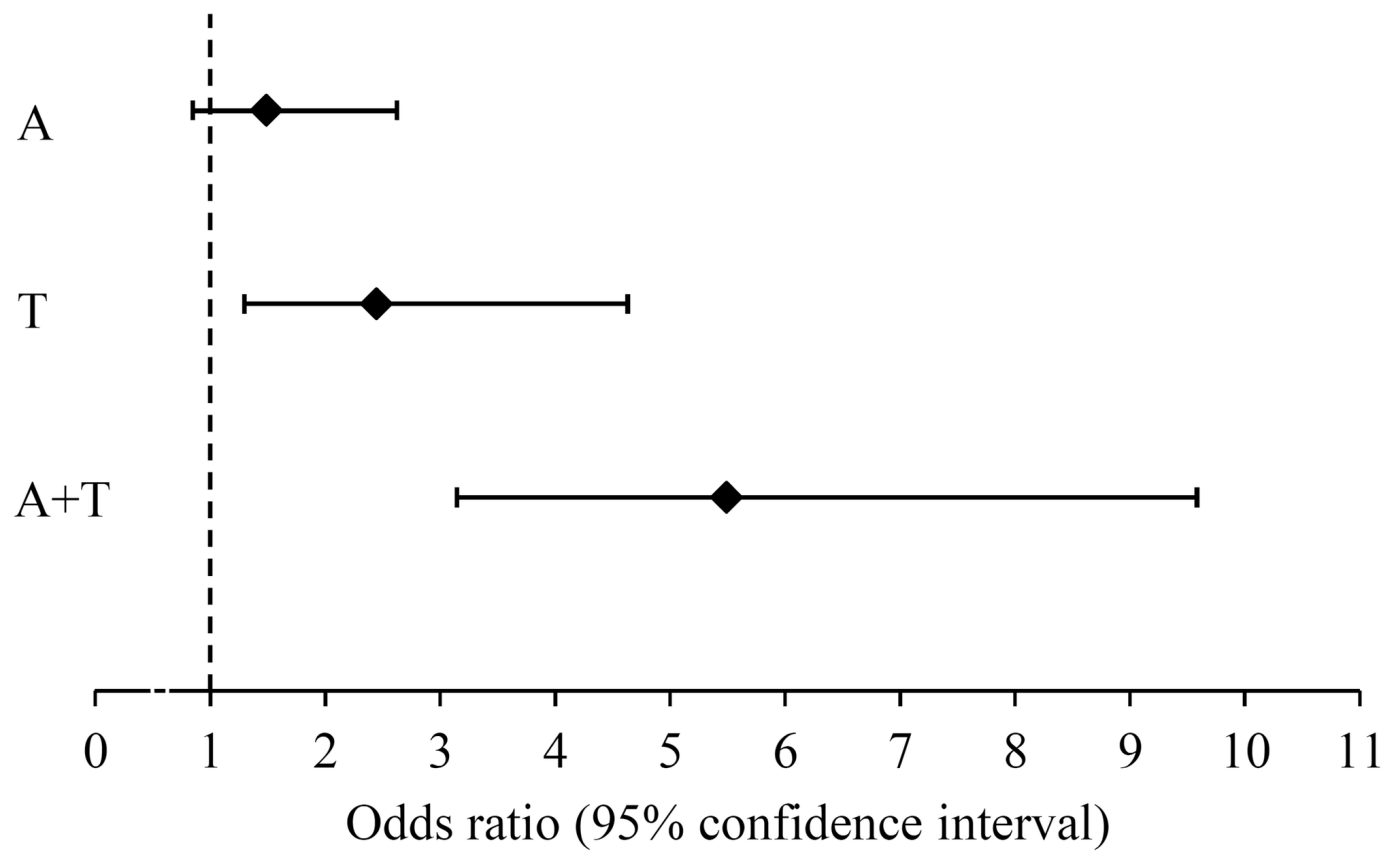
Forest plot of OSA risks for children. *Note*: A = adenoidal hypertrophy; T = tonsillar hypertrophy; A+T = adenotonsillar hypertrophy; OSA = obstructive sleep apnea.

 The OSA risk for subjects with adenoidal hypertrophy only, tonsillar hypertrophy only, and adenotonsillar hypertrophy were first compared to those without adenotonsillar hypertrophy (Figure 2). Subjects with adenotonsillar hypertrophy (OR=5.49; 95% CI 3.15-9.58; p <0.001) and tonsillar hypertrophy only (OR=2.45; 95% CI 1.29-4.63; p=0.006) had a significantly higher OSA risk than children without adenotonsillar hypertrophy. Adenotonsillar hypertrophy in children also increased OSA risk compared to that of children with adenoidal hypertrophy only (OR=3.69; 95% CI 2.08-6.55; p <0.001) or tonsillar hypertrophy only (OR=2.24; 95% CI 1.18-4.28; p=0.014). However, OSA risk did not differed significantly among subjects with adenoidal hypertrophy only and tonsillar hypertrophy only (OR=1.64; 95% CI 0.86-3.16; p=0.136).

### Adenotonsillar size in children in different age groups

The association between adenotonsillar size and AHI in different age groups was investigated (Table 1). A positive association existed between the tonsil grade and an AHI in the toddler group (r=0.34, p=0.029), preschool group (r=0.35, p <0.001), school group (r=0.35, p < 0.001), and the adolescence (r=0.31, p=0.09). Adenoid size and AHI were positively related in the toddler (r=0.37, p=0.019), preschool (r=0.31, p <0.001), and school children (r=0.28, p <0.001), but not in the adolescent group (r=0.11, p=0.368). Table 1 shows the differences in adenoid size and AHI score associations in the toddler, preschool, school children, and the adolescent group.

**Table 1:** The associations between adenotonsillar size and AHI in different age groups.

		Tonsil grade			Adenoid size	
	N	*R*	*P*		*r*	*p*
Age group						
Toddler	42	0.34	0.029*		0.37	0.019*
Preschool	164	0.35	<0.001*		0.31	<0.001*
School	200	0.35	<0.001*		0.28	<0.001*
Adolescence	89	0.31	0.009*		0.11	0.368

Note: *p <0.05 as significant. AHI = apnea/hypopnea index.

### Adenotonsillar size in obese and non-obese children

**Figure 3:**
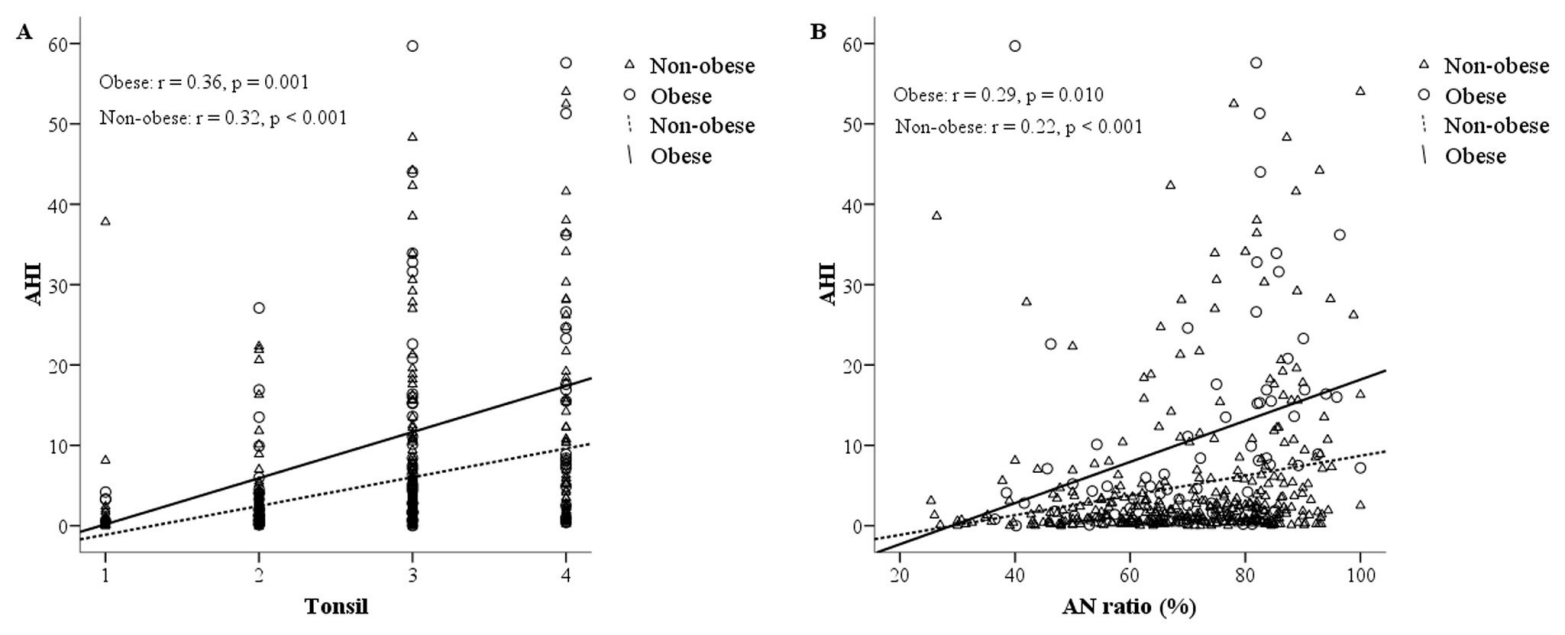
Scatterplot of tonsil grade, AN ratio and AHI in obese and non-obese groups. (**a**) Scatterplot of tonsil grade and AHI in obese and non-obese groups. (**b**) Scatterplot of AN ratio and AHI in obese and non-obese groups. *Note*: **○**: obese; Δ: non-obese. **AN ratio = adenoidal-nasopharyngeal ratio; AHI = apnea/hypopnea index**.

The relationship between tonsil grade and the AHI in the obese and non-obese groups was assessed (Figure 3a). Tonsil grade was positively related to AHI for both obese (r=0.36, p=0.001) and non-obese children (r=0.32, p <0.001). For children with tonsillar hypertrophy, obese children had a higher AHI than non-obese children (Figure 3a). However, tonsil grade and AHI score associations did not differ significantly between obese and non-obese children.

A scatterplot of adenoid size and AHI for obese and non-obese children was drawn (Figure 3b). Adenoid size was positively related to AHI for both obese (r=0.29, p=0.01) and non-obese children (r=0.22, p <0.001). Obese children with adenoidal hypertrophy had a higher AHI than non-obese children (Figure 3b). However, no significant differences existed in the association between adenoid size and the AHI for obese and non-obese children.

### Multivariable regression model

Multivariable logistic regression was applied to analyze the associations between demographics and OSA risk (Table 2). In a multi-logistic regression model, obesity (OR=2.89; 95% CI 1.47-5.68, p=0.002), tonsillar hypertrophy (OR=3.15; 95% CI 2.04-4.88, p <0.001), and adenoidal hypertrophy (OR=1.89; 95% CI 1.19-3.00, p=0.007) significantly increased the risk of OSA for children, whereas age (OR=1.01; 95% CI 0.95-1.08, p=0.703) and gender (OR=1.54; 95% CI 0.97-2.44, p=0.065) was not significantly correlated with pediatric OSA.

**Table 2:** A multivariable logistic regression analysis of the associations between demographics and OSA risk.

Predictor	OR	95% CI	*P value*
Age (per year)	1.01	0.95 - 1.08	0.703
Gender, male	1.54	0.97 - 2.44	0.065
Obese	2.89	1.47 - 5.68	0.002
Tonsillar hypertrophy	3.15	2.04 - 4.88	<0.001
Adenoidal hypertrophy	1.89	1.19 - 3.00	0.007

Note: *p <0.05 as significant. OR = odds ratio; CI = confidence interval.

OSA = obstructive sleep apnea.

## Discussion

This study elucidates the disparities in effects of adenoid size and tonsil size on OSA for different age and adiposity groups. The study by Tagaya et al. [14] observed that the effect of adenoid size on OSA differs between preschool and school children. This study, which further examines the correlation between adenotonsillar size in detailed age categories, found that the effect of adenoid size on OSA decreased in adolescence. Also, adenotonsillar hypertrophy significantly increased OSA risk more than adenoidal or tonsillar hypertrophy alone. The study demonstrates that adenotonsillar hypertrophy is a major element of OSA in children, and the effect of adenotonsillar size on OSA differs for different age categories.

While adenoidal hypertrophy is one of the most important causes of nasal obstruction in children [10,11], methods for evaluating adenoid size remain controversial and unsatisfactory. Many different ways, including lateral radiographs [21,30-33], fiberoptic endoscopy [34,35], and acoustic rhinometry [36], have been advocated as reliable in detecting the adenoidal hypertrophy and its connection to upper airway obstruction. Acoustic rhinometry has been used for cross-sectional area evaluation in the nose. The effect of chronic rhinitis, commonly observed in children with sleep disturbances, may interfere adenoid size assessment [36]. Fiberoptic endoscopy is an accurate diagnostic method that allows examiners to obtain a three-dimensional view of adenoid size. However, children need to cooperate in an endoscopic exam, which is not always possible in children <3 years of age [35]. This study, therefore, used lateral radiographs to explore correlations between adenoid size and OSA for children of different ages.

A lateral cephalometric radiograph is a simple, economical, and reproducible way to measure adenoid size [37,38]. The accuracy of this method has been questioned in view of the fact that these radiographs represent the nasopharynx in only two dimensions [39,40], however, a number of authors have found this examination is practical, and gives satisfactory results for children of all ages [30-33,41-43]. Notably, several radiographic assessment methods have been reported [21,30-33]. Among these, the AN ratio, first described by Fujioka et al., is now the most frequently analyzed radiographic parameter in adenoid size assessment [10,11,15,21,41-43]. Related articles proved the AN ratio is an useful and reliable diagnostic tool [41-43]. Caylakli et al. [41] identified a significant correlation between the AN ratio and endoscopic examination findings. Lertsburapa et al. [42] stated both the AN ratio and endoscopy correlated well with intra-operative exam findings. This study uses the AN ratio because it is an easily applicable and non-invasive method that correctly measures adenoid size in patients of all ages.

 The major goal is to elucidate clearly age-related characteristics of adenotonsillar size and OSA in children. Hypertrophy of the tonsil and/or adenoid is considered the most important risk factor for developing OSA in children [2]. Many investigators are dedicated to unveil the convoluted correlations between adenotonsillar size and childhood OSA [9-11,14,15,44-48], and some authors have even examined the effects of age [14,45]. Valera et al. [45] observed a higher tendency of apnea in young children with adenotonsillar hypertrophy than in older ones. Tagaya et al. [14] noted adenoid size and the apnea index were significantly correlated for preschool children, but not in the school-aged children. Based on previous findings that a stronger correlation exists between adenotonsillar size and OSA in young children than in older children [14,45], this study chose to investigate these correlations for more precise age categories. This study is the first to analyze the correlations between adenotonsillar size and OSA in detailed age groups (i.e., toddler, preschool, school, and adolescent), and obtains several interesting findings. Adenoid size and pediatric OSA are significantly correlated for toddlers, preschoolers, and school-aged children. However, the influence of adenoid size decreases in adolescence. These data are consistent with normal growth patterns of adenoids. The adenoidal-nasopharyngeal space is narrowest at 4.5 years of age, and then the adenoid reaches its greatest size at 7–10 years, when the facial frame develops rapidly [12,14,21]. The adenoidal-nasopharyngeal space gradually decreases until 12 years of age, and sharply diminished from ages 12 to 15 [21]. Interesting, the effects of tonsil size on OSA were similar for all four age groups, indicating that the effects of tonsil size on OSA is still prominent for all children and adolescents. The tonsil growth pattern in children with and without obstructive sleep-disordered breathing (SDB) has been suggested to differ [49]. Kaditis et al. [49] found that young and old children with SDB have similar tonsil size, and age did not affect tonsil size in children with SDB. The analytical results obtained by this study reflect the fact that the influences of adenoid and tonsil size on OSA are intimately correlated with their development and growth phases. These findings also support the concept that from childhood to adulthood, the influences of adenoids decrease, but the influences of tonsils persist in subjects with OSA. This is why surgical modalities for OSA are mainly tonsillectomy with adenoidectomy in children, whereas tonsillectomy with uvuloplasty in adults.

Obesity is an independent risk factor for OSA in children [6,13,22,50]. Adipose tissue deposited around the pharynx and neck, along with hypertrophic adenoids and tonsils, largely contribute to obstructive sleep syndrome in obese children [6,23,50]. Physicians rationally infer that obese children, with equal adenotonsillar size, have a higher AHI than non-obese children. Dayyat et al. [15] stated that the magnitude of adenotonsillar hypertrophy required for any given magnitude of AHI is likely to be smaller in obese children than non-obese children. A similar result was demonstrated in this study (Figure 3). However, in this study, no enough evidence existed that supports the finding that the correlation between adenotonsillar size and OSA is modified by obesity, as described by Dayyat et al.. Care must be taken that in the Dayyat’s study, the sum score of adenotonsillar size, instead of the respective score for adenoid size and tonsil size, was used for data interpretation. This study, on the other hand, demonstrated that adenoid size and tonsil size are both significantly correlated with OSA in obese and non-obese children.

This study further investigated the relative contributions of adenoidal hypertrophy and tonsillar hypertrophy to OSA in children. Based on analytical findings, adenotonsillar hypertrophy increases OSA risk significantly more than adenoidal hypertrophy or tonsillar hypertrphy only. The effect of adenoidal hypertrophy (OR=1.49) and tonsillar hypertrophy (OR=2.45) on OSA risk was amplified when working together (OR=5.49). All these findings suggest that adenoidal hypertrophy and tonsillar hypertrophy have an additive effect on pediatric OSA. Therefore, the treatment strategy for childhood OSA should include both tonsillectomy and adenoidectomy, rather than tonsillectomy or adenoidectomy alone, to achieve optimum postoperative results.

This study has certain limitations. First, it adopted the Fujioka method for adenoid size assessment, rather than measuring all lines in radiographs. Multiple deviant measures of lines may show a discrepancy in the relationships between adenotonsillar size and OSA, although no consensus exists on what are the most useful landmarks in radiographic assessments for adenoid size [10,11]. Second, assessments of adenotonsillar size by radiograph only reflects the nasopharynx in two dimensions. Three-dimensional imaging, including computed tomography or magnetic resonance imaging, although high cost and thus not routinely used, may be more accurate for upper airway evaluations [51,52]. Third, this study could not evaluate precisely nasal conditions as well as the superstructures of the oropharynx and tongue. This study could, therefore, not demonstrate how these structures interact and contribute to pediatric OSA. Fourth, this was a hospital-based study, indicating that patients were recruited from a tertiary medical facility, not from the community. The associations between adenotonsillar size and sleep problems in normal populations require further study.

 In summary, this study delineates the effects of adenotonsillar size on OSA in children with different levels of adiposity and of different age categories. It also investigates contributions of adenoidal hypertrophy and tonsillar hypertrophy to pediatric OSA. Future studies should develop equation models incorporating adenotonsillar size, age, and obesity to predict the surgical outcomes for OSA in children.

## Conclusions

Adenoidal hypertrophy, tonsillar hypertrophy and obesity are major determinants of childhood OSA. Adenotonsillar hypertrophy increases OSA risk significantly more than adenoidal or tonsillar hypertrophy alone. The impact of adenotonsillar size on OSA does not differ between obese and non-obese children, but differ in children of different ages, and the influence of adenoid size decreases in adolescence.
